# The Prevalence of Mood Disorders Among Health and Non-health Undergraduate Students in King Saud University, Riyadh, Saudi Arabia: A Cross-Sectional Study

**DOI:** 10.7759/cureus.51075

**Published:** 2023-12-25

**Authors:** Badr A Alhenaki, Abdulaziz K Alsubait, Mohammed Abuhaimed, Feras O Aljurayyad, Mohammed N Alsubaie, Sajida Agha

**Affiliations:** 1 College of Medicine, King Saud Bin Abdulaziz University for Health Sciences, Riyadh, SAU; 2 Department of Medical Education, King Saud Bin Abdulaziz University for Health Sciences, Riyadh, SAU

**Keywords:** prevalence in saudi arabia, mood disorders, mdd (major depressive disorder), saudi arabia, bipolar disorder

## Abstract

Background: Mood disorders (MDs) are among the most common of all mental health diagnoses, with increasing prevalence and a devastating impact on individuals, families, and the community. This study aimed to estimate the frequency of MDs among health and non-health profession students.

Materials and methods: A cross-sectional survey was conducted on 391 students to estimate the self-reported prevalence of different MDs and to screen for bipolar disorder (BD) using the mood disorder questionnaire (MDQ) and for depressive, anxiety, and stress symptoms using the Depression, Anxiety, and Stress Scale - 21 items (DASS-21).

Results: MDs were reported by 24.9% (n=50) of health profession students and 22.8% (n=31) of non-health profession students. For BD, it affected 35.3% of students in the health profession and 47.4% (n=46) of students without the health profession, although the difference was not statistically significant. The most reported MDs among health and non-health profession students were major depression (4.9% vs. 4.2%), seasonal affective disorder (SAD) (3.3% vs. 2.1%), dysthymia (2.4% vs. 2.8), and BD (2% vs. 2.8%), respectively. None of the observed differences between the two groups were statistically significant. According to DASS-21 scores for health and non-health profession students, severe depressive and severe anxiety symptoms were more common among non-health students (45.1% and 59.3%, respectively) than among health profession students (41.4% and 51.1%, respectively). However, stress was higher among health-related than non-health-related students (19.4% and 18.1%, respectively).

Conclusions: MDs constitute a high burden among university students regardless of their field of study, creating an increased urgency to incorporate ways to promote the mental well-being of students and to manage those with an MD. Further research is needed to identify effective preventive strategies for depression in the future.

## Introduction

Mental health is a fundamental component of health that directly impacts individual, family, and community well-being [1]. Mental and behavioral problems are increasingly being recognized as serious public health concerns. They contribute to 7% of the global burden of disease measured in disability-adjusted life years and 19% of all years lived with disability. Their estimated global prevalence among adults is 13.4% and represents four of the 10 leading causes of disability [2-4].

Mood disorders (MDs) are among the most common mental health diagnoses. The most common are major depressive disorder, dysthymia, seasonal affective disorder (SAD), and bipolar disorder (BD) [5]. Conducting a study on MDs poses several challenges. Conditions falling under this category are quite different, as some of them are transient and others are chronic or recurrent. They are also hardly to be measured or observed directly [5]. People usually have more than one MD during their lives. Non-surprisingly high rates of all types of MDs were reported in previous reviews [5-8].

Major depression is one of the most prevalent MDs and the most commonly investigated one. It involves a depressed mood or a loss of pleasure or interest in activities for long periods of time. Depression is different from regular mood changes and feelings about everyday life. It can affect all aspects of life, including relationships with family, friends, and the community. It can result in or lead to problems at school and work [5,9].

According to the World Health Organization (WHO), the proportion of the global population suffering from depression in 2015 was estimated to be 4.4%. Its prevalence is higher in younger ages [10]. In the United States, the prevalence of adults with a major depressive episode was highest among individuals aged 18-25 (18.6%). A similar figure was reported by epidemiological studies in the Middle East and North Africa regions, where the depression rates demonstrated ranged from 13% to 18% [11-13].

Its importance is gained not only because of its increasing prevalence but also because of its associated disability. In addition, 70% of those with dysthymia may eventually go on to develop major depression. Moreover, approximately 10% of people with major depression eventually develop BD [5]. The Global Burden of Diseases, Injuries, and Risk Factors Study (GBD) 2019 showed that the two most disabling mental disorders were depressive and anxiety disorders, both ranked among the top 25 leading causes of burden worldwide in 2019 [8]. The prevalence of depression over the years showed trends toward an increasing prevalence around the world, and it is estimated that depression will be more of a burden in the future year [5,14].

BD is a serious mental disorder characterized by attacks of depression, hypomania/mania, and mixed episodes, with inter-episodic recovery [15]. It affects 7 billion people in the world with a lifetime prevalence of 1-2.4%. If BD disorder is overlooked, it poses significant morbidity to affected individuals [4,16]. Despite the availability of effective treatments for mental disorders, 75% of affected people in low- and middle-income countries don’t receive treatment. Barriers to effective care include a lack of investment in mental health care, a lack of trained healthcare providers, and social stigma associated with mental disorders [9,17].

The university years are the years when students face physical and emotional challenges related to adolescent changes. BD usually makes its first appearance during university years, when students are still too young to face the additional challenge of major mental disorders [4]. Professional education in the medical field is especially tougher than other streams due to the long and stressful curriculum lasting six years or longer, where students do not engage as often in social and recreational activities. This puts them at a higher risk of mental health conditions like MDs [18,19]. People working in the educational system should consider learners’ mental health and mental adaptability as part of improving the quality of their educational system.

Although depression is the most commonly studied MD, the worldwide rising prevalence and the global challenging stressors facing young adults raise the demand for updated rates for such disabling conditions. Also, other MDs are barely addressed among Saudi students. The hypothesis for the study posits that MDs will be prevalent among both health profession and non-health profession undergraduate students due to increasing global prevalence and the specific challenges faced during university years. The study aimed to screen health and non-health undergraduate students for MD, depression, anxiety, and stress, as well as estimate the self-reported types of MD among students.

## Materials and methods

A cross-sectional survey was conducted to estimate the prevalence of MDs among health and non-health profession students. The study was conducted in health and non-health colleges affiliated with King Saud University, located in Riyadh, Kingdom of Saudi Arabia. All male and female students aged 17 to 28 years were eligible for participation in the study. The estimated number of students studying at the university was 21,550 students/250,000. A non-probability convenience sampling technique was used. A sample of 391 students was enrolled in the study. The sample size was calculated using Epi-Info software (Centers for Disease Control and Prevention, Georgia, USA), and a minimum required sample of 323 students was determined. The calculated sample was based on a 30% prevalence of depression among Umm Al-Qura University students [20] and used a power of 80%, a confidence interval of 95%, and an error of 0.05.

Students who reported ever being diagnosed with MD of any type (BD, depression, major depression, cyclothymia, SAD, persistent depressive disorder (dysthymia), premenstrual dysphoric disorder (PMDD), etc.) are categorized as the group of “mood disorder,” while students who didn’t report any previous diagnosis of any MD are categorized as the group of “no mood disorder.”

University students of different grades were invited to fill out an online or self-administered questionnaire. The questionnaire covered socio-demographic data, including gender, age, specialty, grade point average (GPA), college of study, and students’ grades. Students were also asked about having ever been diagnosed with MD and the type of diagnosis received. Two tools were used to assess the prevalence of MDs among students.

The validated Arabic version of the Mood Disorder Questionnaire (MDQ) is a suitable screening tool for BD, focusing on symptoms of hypomania and mania. These are the mood states that separate BD from other types of depression and MD [21]. It is a self-reporting screening instrument with a Cronbach’s alpha coefficient of 0.83. It consists of 13 yes-or-no items and two other questions, for a total of 15 questions. It takes 5 to 10 minutes to complete. The MDQ questionnaire is a brief instrument that can be used to identify patients most likely to have BD, which is often unrecognized or misdiagnosed at its onset [21].

The items in the tool are based on the Diagnostic and Statistical Manual of Mental Disorders (DSM)-IV criteria for BD. In order to screen positive for possible BD, all three parts of the following criteria should be met: yes to seven or more of the 13 items in question 1, yes to question 2, and moderate or serious problems in question 3.

The validated Arabic version of the Depression, Anxiety, and Stress Scale - 21 items (DASS-21) is a set of three self-report scales designed to measure the emotional states of depression, anxiety, and stress [22]. The DASS is a reliable tool with an acceptable range of internal consistency and concurrent validity. Based on the referenced study by Terkawi et al. [23], the internal consistency of the tool was reported to be 0.83 (95% confidence interval (CI): 0.79-0.88). The study also demonstrated a strong correlation between the tool and the generalized anxiety disorder score, indicating concurrent validity.

The study was approved by the Ethics Committee of King Abdullah Medical Research Center (approval number: 1769/23). Permissions from the deanships of the colleges where the participants were enrolled were obtained before conducting the study. The study objectives were clearly explained to prospective participants. Only students who gave their consent to participate were included in the study and asked to fill out the questionnaires in their free time. A Google form (Google LLC, California, USA) was created, and a link was emailed to all students' official email addresses. Students’ anonymity, confidentiality, and privacy were assured. The respondents were informed that they had the freedom to withdraw from the research study at any time without any consequence. We sent two reminders to students at a one-month interval.

All the responses were entered in the Excel sheet (Microsoft Corporation, Washington, USA). Statistical analysis was performed using SPSS Statistics version 20.0 (IBM Corp. Released 2011. IBM SPSS Statistics for Windows, Version 20.0. Armonk, NY: IBM Corp.). For bivariate analysis, the chi-square test was used for categorical variables and Fisher's exact test when the chi-square test was not valid. In bivariate analysis, a p-value of less than 0.05 was considered statistically significant.

## Results

A total of 391 participants were included in this study. About two-thirds of the respondents were males (67%, n=261). Respondents’ mean age was 20.5 with a standard deviation of 1.5. Around half (51%, n=196) of the respondents had a GPA greater than 4.5, and the lowest percentage of respondents (4%, n=15) had a GPA <3.5. The highest representation of health field students was from the faculty of pharmacy and medicine (45% (n=102), 27% (n=61), respectively), and the rest came from the other faculties. The non-health faculties were represented mainly by engineering students (64% (n=109)). Most students were enrolled in the fourth through sixth years (37%, n=144), followed by the first (24%, n=92) and third (22%, n=86) years. According to students’ reports of being diagnosed with MD of any type, about one in four students (24%, n=92) had been previously diagnosed with an MD (Table 1).

**Table 1:** Study respondents' characteristics Categorical data presented n (%), numerical presented mean±SD GPA: grade point, MD: mood disorder

Characteristic (N=391)		n (%)
Gender	Male	261 (67)
	Female	130 (33)
Age (in years)	Mean±SD	20.5±1.5
Field of study	Health	245 (63)
	Non-health	143 (37)
Academic year	First	92 (24)
	Second	67 (17)
	Third	86 (22)
	Fourth-sixth	144 (37)
College (health)	College of medicine	61 (27)
	College of pharmacy	102 (45)
	College of nursing	17 (8)
	College of dentistry	10 (4)
	Other	36 (16)
College (non-health)	College of engineering	109 (64%)
	College of business administration	24 (14%)
	College of arts	32 (19%)
	Other	5 (3%)
Student GPA (N=388)	>4.5	196 (51%)
	4-4.5	122 (31%)
	3.5-4	55 (14%)
	<3.5	15 (4%)
	Mean±SD	1.7±0.85
MD (N=339)	No MD	258 (76%)
	MD	81 (24 %)

**Figure 1:**
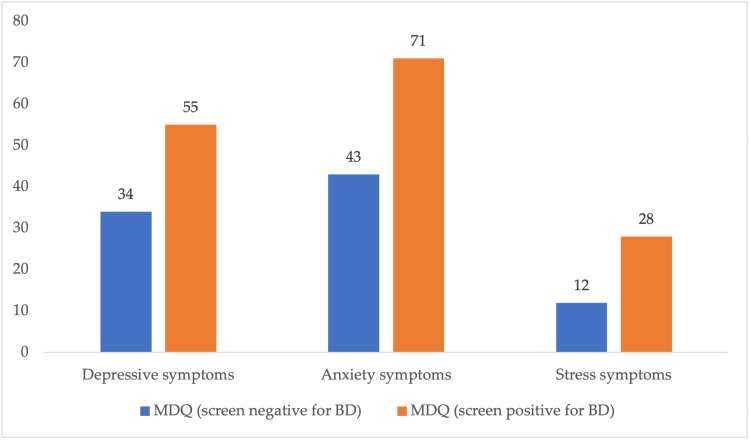
Severe depression, anxiety, and stress among students who screened positive and negative for BD according to the MDQ scale Data presented in the form of number (%) MDQ: mood disorder, BD: bipolar disorder

Based on their self-report of receiving previous diagnoses of any MD, students were classified into two groups: the “mood disorder” group and the “no mood disorder” group. Table 2 shows the distribution of respondents according to some personal characteristics and their MD group. Only 216/219 students responded to the DASS-21 scale questions, and the missing responses stand for the fewer numbers of students in Table 2. Chi-squared test results appeared in the table to show an association between the independent variables and the MD. Females showed a higher percentage of MDs than males (67% vs. 37%, respectively), with insignificant results for chi-square (p=0.661). The highest frequency of MD was encountered among students with GPAs of >4.5 (44.4%) followed by students with GPAs of 4-4.5 (28.4%); however, this association was non-significant (p=0.196). Students in the health fields appeared to have a higher percentage of MDs (61.7%) and only 38.3% of students in the health fields, with non-significant association (p=0.661). With regards to the association of MD history and current affection with depression, anxiety, and stress symptoms, students with MD were more encountered among the severe/extremely severe group for depression (p<0.001), anxiety (27.2%, p<0.001), and stress (25.6%, p=0.007). This is further illustrated in Figure 1, where severe depression, anxiety, and stress were significantly higher among students who screened positive and negative for BD according to the MDQ scale.

**Table 2:** The distribution of respondents according to gender, GPA, field of study, academic year, depression, anxiety, stress, and their previous diagnosis with MD DASS: Depression, Anxiety, and Stress. *** p-value <0.001, ** p-value <0.01, * p-value <0.05, p-value <0.1; percentages in brackets; categorical data presented as n (%), numerical data presented as mean±SD MDQ: Mood Disorder Questionnaire. *** p-value <0.001, ** p-value <0.01, * p-value <0.05, p-value <0.1; percentages in brackets; categorical data presented as n (%), numerical data presented as mean±SD MD: mood disorder, GPA: grade point average

Label	Levels	no MD	MD	p-value
Gender	Male	185 (71.7)	51 (63.0)	0.661
	Female	48 (28.3)	32 (37.0)	
What is your GPA	>4.5	128 (49.6)	36 (44.4)	0.196
	4-4.5	87 (33.7)	23 (28.4)	
	3.5-4	33 (12.8)	18 (22.2)	
	<3.5	10 (3.9)	4 (4.9)	
What is your field of study?	Health	151 (59.0)	50 (61.7)	0.661
	Non-health	105 (41.0)	31 (38.3)	
Academic year	First	49 (19.0)	15 (18.5)	0.884
	Second	48 (18.6)	14 (17.3)	
	Third	65 (25.2)	18 (22.2)	
	Fourth-sixth	96 (37.2)	34 (42.0)	
Depressive symptoms (DASS-21)	Normal	45 (26.2)	2 (5.1)	<0.001***
	Mild-moderate	64 (37.2)	8 (11.1)	
	Severe-extremely severe	63 (36.6)	29 (74.4)	
Anxiety symptoms (DASS-21)	Normal	41 (23.7)	1 (2.7)	<0.001***
	Mild-moderate	49 (28.3)	5 (13.5)	
	Severe-extremely severe	83 (48.0)	31 (83.8)	
Stress symptoms (DASS-21)	Normal	50 (28.7)	2 (5.1)	0.007**
	Mild-moderate	95 (54.6)	27 (69.2)	
	Severe-extremely severe	29 (16.7)	10 (25.7)	
Bi-polar symptoms (MDQ)	Screen positive	75 (37.5)	26 (56.5)	0.018*
	Screen negative	125 (62.6)	20 (43.5)	

**Figure 2:**
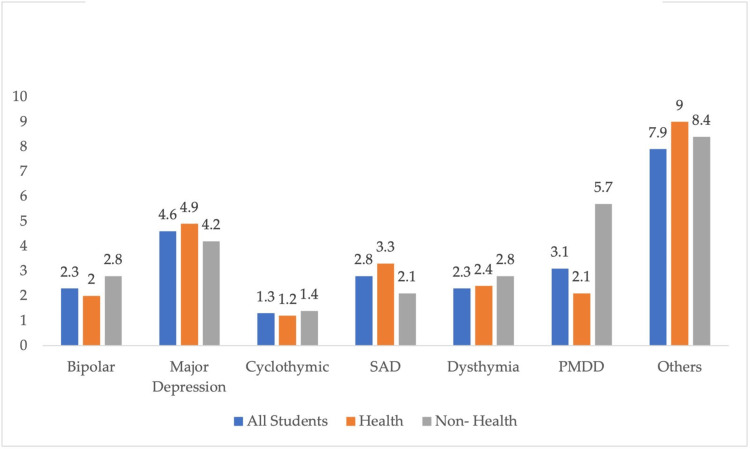
Frequency of the different types of MDs disorders according to the field of study Data presented in the form of number (%) SAD: seasonal affective disorder, PMDD: premenstrual dysphoric disorder

Table 3 and Figure 2 show the frequency of the different types of MDs and their associations with the type of college they study, whether health or non-health. Major depression was the most common MD among the studied sample (4.6%, n=18), followed by SAD (2.8%, n=11). Major depression and SAD were more common among health students than non-health profession students (4.9% vs. 4.2% and 3.3% vs. 2.1%), while other conditions were more common among non-health profession students, with bipolar diagnosis and dysthymia being the most common (2.8% vs. 2% and 2.8% vs. 2.4%). PMDD was the most common disorder among non-health profession students. Only 255 students responded to the MDQ scale questions. On the MDQ scale, bipolar symptoms were more common among non-health profession students (47.5% vs. 35.3%), with bare significance (p=0.055). Severe depression (45.1% vs. 41.4%) and anxiety symptoms (59.3% vs. 51.1%) were more common among non-health profession students than health profession ones. However, severe stress symptoms were slightly more common among health profession students (19.4% vs. 18.1%). However, all those associations were not significant.

**Table 3:** The distribution of respondents according to MD types, depression, anxiety, stress, and field of study DASS: Depression, Anxiety, and Stress. *** p-value <0.001, ** p-value <0.01, * p-value <0.05, p-value <0.1; percentages in brackets; categorical data presented as n (%), numerical data presented as mean±SD MDQ: Mood Disorder Questionnaire. *** p-value <0.001, ** p-value <0.01, * p-value <0.05, p-value <0.1; percentages in brackets; categorical data presented as n (%), numerical data presented as mean±SD (a) Fisher's exact test, (b) female respondents only. BD: bipolar disorder, SAD: seasonal affective disorder, PMDD: premenstrual dysphoric disorder

Label	Levels	Health profession n (%)	Non-health profession n (%)	p-value
BD	Yes	5 (2)	4 (2.8)	0.438^*(a)^
	No	240 (98.0)	139 (97.2)	
Major depression	Yes	12 (4.9)	6 (4.2)	0.751
	No	233 (95.1)	137 (95.8)	
Cyclothymic	Yes	3 (1.2)	2 (1.4)	0.608^(a)^
	No	242 (98.8)	141 (98.6)	
SAD	Yes	8 (3.3)	3 (2.1)	0.373^(a)^
	No	237 (96.7)	140 (97.9)	
Dysthymia	Yes	6 (2.4)	4 (2.8)	0.537
	No	239 (97.6)	139 (97.2)	
PMDD ^(b)^	Yes	2 (2.1)	2 (5.7)	0.574^(a)^
	No	92 (97.9)	33 (94.3)	
Bi-polar symptoms (MDQ)	Screen positive	55 (35.3)	46 (47.4)	0.055
	Screen negative	101 (64.7)	51 (52.6)	
Depressive symptoms (DASS-21)	Normal	33 (24.8)	15 (18.3)	0.537
	Mild-moderate	45 (33.8)	30 (36.6)	
	Severe-extremely severe	55 (41.4)	37 (45.1)	
Anxiety symptoms (DASS-21)	Normal	27 (20.3)	16 (19.8)	0.417
	Mild-moderate	38 (28.6)	17 (21.0)	
	Severe-extremely severe	68 (51.1)	48 (59.3)	
Stress symptoms (DASS-21)	Normal	35 (26.1)	18 (21.7)	0.684
	Mild-moderate	73 (54.5)	50 (60.2)	
	Severe-extremely severe	26 (19.4)	15 (18.1)	

**Figure 3:**
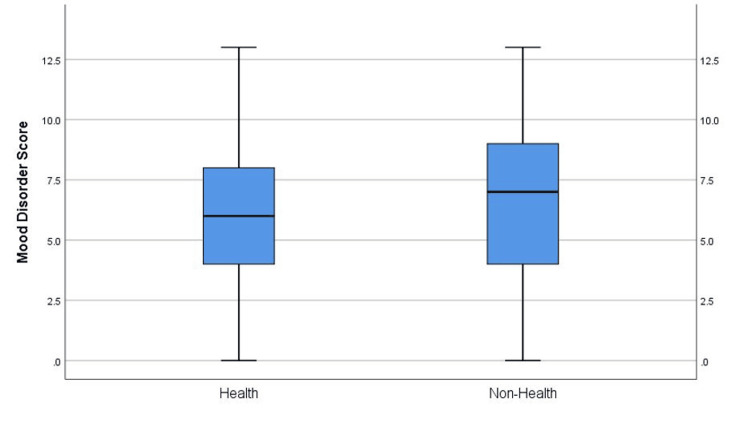
Boxplot for summary statistics of the MD score grouped by field of study Data presented in the form of number

Figure 3 is a box plot that provides the summary of statistics for respondents (who responded to the MD questions on the MDQ) according to the field of study (health (n=156) and non-health studies (n=97)). The mean score for MDs among students who study health is 5.910, and the median is 6.0. Additionally, the mean score for MDs among non-health field students is 6.72, and the median is 7.0. The standard deviation in the two categories is almost equal (3.2456) and the range is also the same (13%).

## Discussion

The present study further emphasized the burden of MD among Saudi students, where one in four students (24%) had a diagnosis of MD of any type, with major depression being the most common. This figure is not far from or even lower than the global prevalence (28.9%) of depression among students in the health and non-health professions [24]. However, our figure was less than that reported by El Kot et al. [20], which studied depression only. This difference could mainly be attributed to the methodological differences between the different studies.

Surprisingly, the self-reported prevalence of major depression among students was 4.6%, while using the DASS-21 questionnaire, more than 80% of students showed depressive symptoms, with more than 40% of them showing severe to extremely severe symptoms regardless of the type of college they are enrolled in. Additionally, screening for BD using MDQ showed that 35.3% of students in the health profession and 47.4% of students in the non-health profession screened positive for BD. Furthermore, stress and anxiety symptoms were also common among about 80% of the students.

It is generally thought that health students might suffer from difficulties and stresses in their field of study that make them more prone to emotional problems and MDs, which, if not solved, might predispose students to further stresses [25,26]. However, our study didn’t show significant differences in the prevalence among health and non-health profession students. This finding is logical and generally accepted that many other non-health profession students also suffer from similar stresses and difficulties in their studies, such as engineering students. Our finding also goes with the finding reported by a meta-analysis, which identified no significant difference in the global prevalence of depression between medical and non-medical students [24].

Unlike previous studies conducted in Saudi Arabia [20,27], our study failed to identify gender differences in the prevalence of self-reported MDs. Methodological differences used to assess depression could stand behind this contradiction.

The high figures mentioned above for MD burden and the similar rates of the different MDs among the health and non-health profession groups draw attention to the high burden of MDs among all university students (including males and females), calling for serious responses and actions to be taken where neither group is discounted. Broader-scope studies using different, more sensitive, and accurate tools are needed to screen and diagnose university students early and act accordingly. In addition, students' help- and treatment-seeking behaviors should be investigated to understand and decide on appropriate treatment and intervention options as early as possible. Students may need encouragement to seek help and comply with treatment. In addition, more studies are needed to understand and investigate the factors that influence and correlate with different MDs. Such factors could be socio-culturally related to family and community environments or the academic learning environment.

Though our study has provided valuable information concerning the burden of MDs among university students in Saudi Arabia, several limitations were faced. First, because the survey was conducted online, we cannot guarantee the accuracy of the results. In addition, online sampling tends to be convenience sampling, limiting study generalizability. Second, our data was obtained from only one university in a short period of time, and many potential students were excluded from the study. Third, the study did not look at the relationship between mental health and socio-demographic and clinical factors. Fourth, missing students’ responses for the MDQ and DASS-21 scale limited testing of their association with other variables.

## Conclusions

The current study findings indicate that MDs constitute a high burden among university students, regardless of their field of study. They also denote that students with MDs suffer emotional problems that are not resolved, implying either no or inadequate treatment. These findings create an increased urgency to incorporate ways to promote the mental well-being of students and to manage those with MDs. University mental health services should expand to include both health and non-health profession students. Addressing MDs and emotional problems among students through the establishment of psychosocial networks or support systems is a priority. It is suggested that a multi-center investigation with a larger number of participants could aid in the identification of determinants and causes of MDs, especially those of low prevalence. Further research is needed to identify the determinants and causes of MD among university students and effective strategies to address those problems.
